# RNAemia Corresponds to Disease Severity and Antibody Response in Hospitalized COVID-19 Patients

**DOI:** 10.3390/v12091045

**Published:** 2020-09-18

**Authors:** Kirsten Alexandra Eberhardt, Charlotte Meyer-Schwickerath, Eva Heger, Elena Knops, Clara Lehmann, Jan Rybniker, Philipp Schommers, Dennis A. Eichenauer, Florian Kurth, Michael Ramharter, Rolf Kaiser, Udo Holtick, Florian Klein, Norma Jung, Veronica Di Cristanziano

**Affiliations:** 1 Department of Tropical Medicine, Bernhard Nocht Institute for Tropical Medicine and I. Department of Medicine, University Medical Center Hamburg-Eppendorf, 20251 Hamburg, Germany; k.eberhardt@bnitm.de (K.A.E.); florian.kurth@bnitm.de (F.K.); ramharter@bnitm.de (M.R.); 2 Department I of Internal Medicine, Medical Faculty and University Hospital Cologne, University of Cologne, 50937 Cologne, Germany; charlotte.meyer-schwickerath@uk-koeln.de (C.M.-S.); clara.lehmann@uk-koeln.de (C.L.); jan.rybniker@uk-koeln.de (J.R.); philipp.schommers@uk-koeln.de (P.S.); dennis.eichenauer@uk-koeln.de (D.A.E.); udo.holtick@uk-koeln.de (U.H.); norma.jung@uk-koeln.de (N.J.); 3 Institute of Virology, Faculty of Medicine and University Hospital of Cologne, University of Cologne, 50935 Cologne, Germany; eva.heger@uk-koeln.de (E.H.); elena.knops@uk-koeln.de (E.K.); rolf.kaiser@uk-koeln.de (R.K.); florian.klein@uk-koeln.de (F.K.); 4 German Center for Infection Research (DZIF), Partner Site Bonn-Cologne, 50937 Cologne, Germany; 5 Center for Molecular Medicine Cologne, University of Cologne, 50937 Cologne, Germany; 6 Center of Integrated Oncology ABCD, University Hospital of Cologne, 50937 Cologne, Germany; 7 Intensive Care Program, Department I of Internal Medicine, University Hospital of Cologne, 50937 Cologne, Germany

**Keywords:** SARS-CoV-2, humoral response, seroconversion, dynamics, viral load, blood, viremia, antibodies

## Abstract

Severe acute respiratory syndrome coronavirus 2 (SARS-CoV-2) represents a global health emergency. To improve the understanding of the systemic component of SARS-CoV-2, we investigated if viral load dynamics in plasma and respiratory samples are associated with antibody response and severity of coronavirus disease 2019 (COVID-19). SARS-CoV-2 RNA was found in plasma samples from 14 (44%) out of 32 patients. RNAemia was detected in 5 out of 6 fatal cases. Peak IgG values were significantly lower in mild/moderate than in severe (0.6 (interquartile range, IQR, 0.4–3.2) vs. 11.8 (IQR, 9.9–13.0), adjusted *p* = 0.003) or critical cases (11.29 (IQR, 8.3–12.0), adjusted *p* = 0.042). IgG titers were significantly associated with virus Ct (Cycle threshold) value in plasma and respiratory specimens ((ß = 0.4, 95% CI (confidence interval, 0.2; 0.5), *p* < 0.001 and ß = 0.5, 95% CI (0.2; 0.6), *p* = 0.002). A classification as severe or a critical case was additionally inversely associated with Ct values in plasma in comparison to mild/moderate cases (ß = −3.3, 95% CI (−5.8; 0.8), *p* = 0.024 and ß = −4.4, 95% CI (−7.2; 1.6), *p* = 0.007, respectively). Based on the present data, our hypothesis is that the early stage of SARS-CoV-2 infection is characterized by a primary RNAemia, as a potential manifestation of a systemic infection. Additionally, the viral load in plasma seems to be associated with a worse disease outcome.

## 1. Introduction

The severe acute respiratory syndrome 2 coronavirus (SARS-CoV-2) pandemic represents a global health emergency [1]. Although the pathogenicity of SARS-CoV-2 is lower in comparison to other zoonotic coronaviruses, such as SARS-CoV (severe acute respiratory syndrome coronavirus) and MERS-CoV (middle east respiratory syndrome coronavirus), the virus is highly contagious and asymptomatic carriers are suspected of playing a crucial role in the spread of the pandemic [2,3].

Evidence that SARS-CoV-2 RNA was detected in blood samples of asymptomatic blood donors raised the issue about the safety of blood transfusions [4,5]. However, the systemic spread of SARS-CoV-2 still represents a poorly considered aspect of this novel emerging viral agent [6].

As described in influenza viruses, a viremic phase is a known event in viral respiratory diseases, repeatedly observed in new pandemic strains. Virus isolation was successfully performed from serum samples of infected patients in different fatal cases of avian influenza virus, evidencing the clinical importance of this topic [7,8]. Both transneuronal and hematogenous routes were suggested in cases of CoV infections of the nervous system [9]. For SARS-CoV, a viremic spread, as well as the capacity of the virus to infect extrapulmonary tissues, including immune blood cells, kidney, and brain have been reported [10]. Indeed, extrapulmonary manifestations of SARS-CoV-2 have been described as well [11]. Reports of acute kidney invasion of the virus and cardiac manifestations suggest the ability of the virus to invade other organs [12,13].

The aim of the present study was to provide new evidence about detection rate and a temporal profile of SARS-CoV-2 RNAemia compared to the detection in respiratory samples in hospitalized coronavirus disease 2019 (COVID-19) patients. Furthermore, the viral loads in both compartments were analyzed in relation to antibody response and the severity of disease.

## 2. Materials and Methods

### 2.1. Patients

Consecutive patients with laboratory confirmed SARS-CoV-2 treated at the University Hospital of Cologne, Germany, between 2 March 2020 and 27 May 2020, were included in the present study. Initial diagnosis was confirmed by real-time PCR (RT-PCR) of pharyngeal samples (pharyngeal swabs, tracheal or bronchial secretions) and routinely repeated during the course of disease. Additionally, RT-PCR of plasma, as well as SARS-CoV-2 antibody determination was routinely performed. Further, demographic and relevant clinical information was collected. This study was approved by the Institutional Review Board of the University of Cologne (20–1254).

### 2.2. Molecular Detection of SARS-CoV-2 RNA in Respiratory and Plasma Samples

Respiratory samples were analyzed either by Cobas^®^ SARS-CoV-2 Test targeting the regions *E*-gene and *ORF* (open reading frame)-1a/b on the automated Cobas^®^ 6800 (Roche Diagnostics, Mannheim, Germany), by the RealStar^®^ SARS-CoV-2 RT-PCR Kit 1.0 targeting *E* gene and *S* gene (Altona Diagnostics, Hamburg Germany), or by the LightMix^®^ SarbecoV *E*-gene plus EAV control (TIB Molbiol, Berlin, Germany). In this last case, the second target *RdRP*-gene (RNA dependent RNA polymerase, parallel) by LightMix^®^ Modular Wuhan CoV *RdRP*-gene (TIB Molbiol) or the *N*-gene (inhouse primer sets in multiplex PCR) were used. The two latter assays were carried out on LightCycler^®^ 480 (Roche Diagnostics). Sample preparation was performed using the MagNA Pure 96 System (Roche Diagnostics). All plasma samples were tested by Cobas^®^ SARS-CoV-2 Test on the automated Cobas^®^ 6800 (Roche Diagnostics) [14].

Used kits passed the recently distributed quality management (QM) procedure from Instand [15].

### 2.3. Detection of SARS-CoV-2 IgG

The Euroimmun anti-SARS-CoV-2 ELISA for immunoglobulin class G targeting the S1 domain of the spike protein was used for serological testing (Euroimmun Diagnostik, Lübeck, Germany). According to manufacturer’s recommendations, IgG values were interpreted as positive if the ratio (extinction value of patient sample/extinction value of calibrator) was ≥1.1.

### 2.4. Statistical Analysis

Continuous variables were expressed as median (interquartile range, IQR) and compared using the Kruskal–Wallis Test. In case of a significant difference, pairwise comparisons between group levels were performed and adjusted for multiple comparisons using the Benjamini and Hochberg method [16]. Categorical variables were compared using either the χ^2^ test or the Fisher exact test, as appropriate. A random intercept model determining patients as random effects was used to assess the association between covariates and the continuous outcome SARS-CoV-2 RNA RT-PCR Ct (Cycle threshold) value. Statistical significance was determined at α = 5%. In figures, negative RT-PCR Ct values were displayed as a value of 42, which is outside of the detection area. Statistical analyses were performed using R (v3.6.3, R Foundation for Statistical Computing, Vienna, Austria).

## 3. Results

### Patient Characteristics

Thirty-two patients treated for COVID-19 at the University Hospital of Cologne, Cologne, Germany, were included in the present analysis. Based on current literature, six patients (19%) were classified as mild/moderate disease severity, 14 (44%) as severe, and 12 patients (37%) as critically ill cases (Table 1 and Supplementary Table S1) [17,18,19].

Among all three groups, no differences were detected for lymphocyte and leucocyte counts at the time of first positive SARS-CoV-2 RT-PCR test result in respiratory specimen after hospital admission. In contrast, C-reactive protein (CRP) and interleukin 6 (IL-6) were significantly higher in critically ill individuals as compared to patients with severe, mild or moderate disease at the same timepoint. Coexisting disorders varied from cardiac diseases to malignancies and were present in 81% of patients. Six persons (19%) died during observation time (Table 1 and Supplementary Table S1).

**Figure 1:**
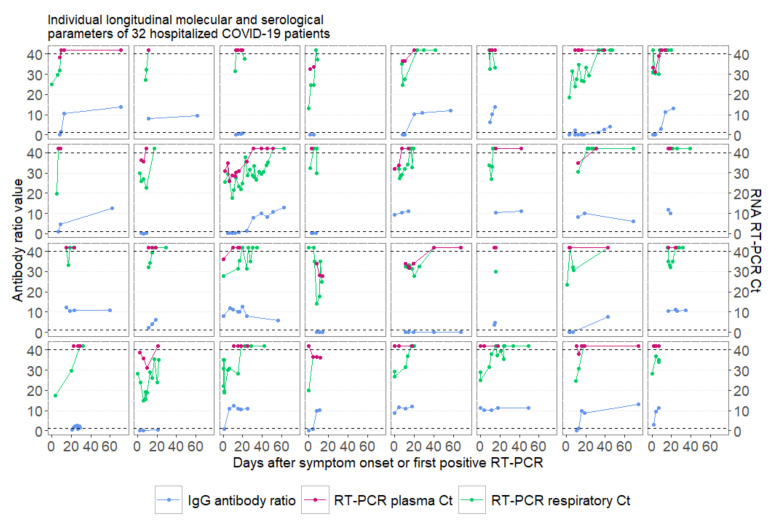
Individual longitudinal development of virologic and serological results of 32 patients with coronavirus disease 2019 (COVID-19) infection during hospital admission. Negative PCR results are displayed above the detection Ct (Cycle threshold) of 40. Dashed horizontal lines display the detection threshold of severe acute respiratory syndrome coronavirus 2 (SARS-CoV-2) RNA Ct values and the antibody cut-off of seroconversion for the assay used. RT-PCR, real-time PCR.

Overall, 117 respiratory and 112 plasma samples from these 32 patients were tested for SARS-CoV-2 by RT-PCR during observation time (Figure 1). Virus RNA was detected in plasma samples from 14 patients (44%). Of note, in five out of six lethal cases, SARS-CoV-2 RNA was present in plasma (Table 1). The medium time from initial symptoms to last positive plasma PCR result before turning negative was 14 days (IQR, 10–15).

**Figure 2:**
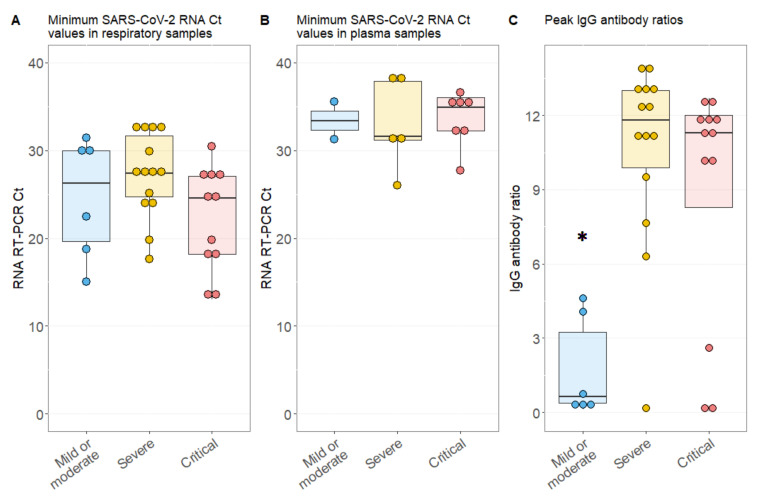
Molecular and serological parameters according to disease severity in COVID-19 patients. (**A**) Lowest detected SARS-CoV-2 RNA RT-PCR Ct value in respiratory samples per patient; (**B**) nadir SARS-CoV-2 RNA RT-PCR plasma Ct value per patient; (**C**) peak IgG value per patient; * *p* < 0.05; error bars, 95% confidence interval.

Although peak viral loads in respiratory samples were higher compared to those in plasma samples, represented by lower SARS-CoV-2 Ct values (Figure 2A,B), nadir Ct values in respiratory samples were not different among patients with or without RNAemia (24.6 (IQR, 18.2–27.6) vs. 27.0 (IQR, 23.5–31.4), *p* = 0.071, Supplementary Figure S1). Only two individuals from the severe and critical severity group had plasma Ct values below 30, indicating that plasma viral loads were generally low (Figure 2B).

Peak IgG values during observation time were significantly lower in patients with a mild or moderate disease course than in severe (0.6 (IQR, 0.4–3.2) vs. 11.8 (IQR, 9.9–13.0), adjusted *p* = 0.003) or critical ill cases (11.29 (IQR, 8.3–12.0), adjusted *p* = 0.042). In some mild cases, the lack of detection of seroconversion was linked to immunological disorders or early discharge of individual subjects (Figure 2C and Supplementary Table S1). Interestingly, the ability to produce a sufficient humoral antibody response was not different for patients with or without RNAemia as measured with peak IgG values per patient (10.7 (IQR, 0.3–12.9) vs. 11.1 (IQR, 5.1–11.9), *p* = 0.985, Supplementary Figure S1).

**Figure 3:**
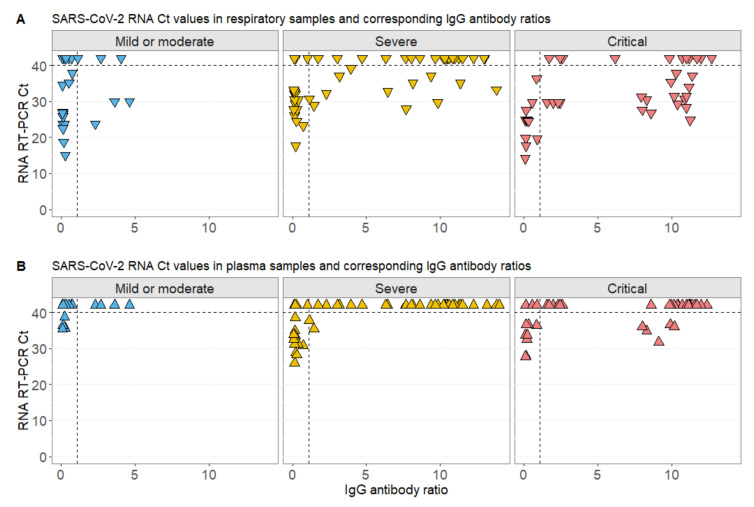
SARS-CoV-2 Ct values in relation to IgG ratios according to COVID-19 severity. (**A**) Relation of SARS-CoV-2 respiratory Ct values with IgG values at the same day ± 2 days; (**B**) relation of SARS-CoV-2 plasma Ct values with IgG values at the same day ± 2 days in patients with mild or moderate, severe, or critical COVID-19 disease severity. Negative PCR results are displayed above the detection Ct of 40. Dashed horizontal lines display the detection threshold of SARS-CoV-2 RNA Ct values and the antibody cut-off of seroconversion for the assay used.

Detectable virus RNA, displayed as Ct values below 40, was commonly present in respiratory samples of patients with high IgG titers at the same time for all disease severity levels (Figure 3A and Supplementary Figure S2). On the other hand, RNAemia was almost exclusively detected in patients that were not seroconverted at this timepoint. Only in five samples from four critically ill cases (including three fatalities) was virus RNA detected in plasma samples despite high IgG (Figure 3B and Supplementary Figure S2).

In a random intercept model with patient as random effect, the IgG titer was significantly associated with SARS-CoV-2 Ct values in samples from the respiratory tract (ß = 0.5, 95% CI (0.2; 0.6), *p* = 0.002) after adjustment for other parameters, including sex, age, days after onset of symptoms, and disease severity (Table 2). This finding indicated that SARS-CoV-2 RNA decreased with specific antibody production. Likewise, in a similar mixed model for the continuous outcome SARS-CoV-2 Ct value in plasma samples, IgG titer was significantly associated with the Ct value (ß = 0.4, 95% CI (0.2; 0.5), *p* < 0.001). In contrast to the model above, the disease severity was additionally associated with the detected RNA Ct values in plasma (ß = −3.3, 95% CI (−5.8; 0.8), *p* = 0.024 and ß = −4.4, 95% CI (−7.2; 1.6), *p* = 0.007, respectively) (Table 2).

## 4. Discussion

Despite intensive research on COVID-19, the detection of SARS-CoV-2 in blood samples has been marginally investigated so far. At the beginning of the pandemic, data from China indicated positive detection rates in the blood ranging between 1% and 41% [6,20,21]. Differently from the data published by Wölfel et al. [22] from nine patients with mild SARS-CoV-2 infection, 14 (44%) out of 32 hospitalized patients in our cohort were detected to be SARS-CoV-2 RNA positive in plasma. A recently published work by Veyen et al. reported a detection rate of 74.1% by using droplet-based digital PCR, representing one of the highest detection rates described so far [23].

As previously reported, other respiratory viral agents were detected in blood [24]. Rhinovirus C RNAemia was identified in 10% of hospitalized children affected from pneumonia [25]. Respiratory syncytial virus (RSV) was identified in immune blood cells from children affected from bronchiolitis [26]. Data from the SARS-CoV epidemic reported a positive detection rate of up to 79% in plasma samples collected within the first three days after the onset of fever and plasma samples were proposed for early diagnosis of SARS. Furthermore, a high viral load in blood was related to a worse outcome [27,28,29]. Also in case of new pandemic influenza strains, a viremic phase was shown to represent a relevant aspect in the clinical course of infection [30]. In 1963, Naficy et al. isolated influenza A (H2N2) virus from the blood of a patient five days after onset of symptoms [31]. Khakpour et al. described a viremic episode 12 h before onset of symptoms of Asian influenza virus [32]. Poliakova et al. detected the same virus in 11 out of 63 patients during the first days of symptoms [33]. In case of influenza A (H1N1) virus, the viremia was associated with disease severity [34,35]. Influenza A (H5N1) virus RNAemia was detected in nine out of 16 infected patients and linked with a general high viral burden and poor prognosis [8]. Likewise, in the present cohort of COVID-19 patients, five out of six fatal cases were detected positive for SARS-CoV-2 RNA in plasma during the course of disease. Moreover, as the only fatal case without RNA detection in plasma was already admitted with a high IgG titer, a positive detection in plasma prior to admission might be conceivable. Although univariate comparisons did not illustrate differences in viral load peaks in patients with mild compared to more severe ill patients, the applied mixed model revealed a significant association of viral load in plasma and disease severity of COVID-19. Accordingly, Chen et al. detected RNAemia exclusively in the critically ill group (5/17) with no detection in the less severe cases (0/31) [19].

As already reported by previous studies, patients with severe or critical COVID-19 displayed a more intense humoral response than moderate and mild cases [36]. Our analysis also revealed a significant inverse association of humoral response with SARS-CoV-2 RNAemia. In case of MERS-CoV, Corman et al. reported an inverse correlation between viremia and humoral response, even if viral RNA and neutralizing antibodies were also simultaneously detected [37]. The statistically significant inverse association of RNAemia with the specific IgG antibody response shown in the present study may suggest the potential of the humoral response to control the systemic spread of SARS-CoV-2.

In samples obtained from the respiratory tract, the viral load was also inversely associated with the development of specific SARS-CoV-2 IgG. Additionally, the negation of PCR results correlated with the duration of infection, as already described [38]. Published data indicated that Ct values from diagnostic respiratory samples and the duration of disease are important elements to assess the infectivity of patients [39].

This study has some important limitations. As data derived from routine laboratory samples and patients were heterogenous with regard to duration and severity of the disease, time intervals of testing and follow-up times varied between them. Additionally, we did not calculate sample sizes for our analysis as we used available routine laboratory data for a retrospective analysis.

## 5. Conclusions

Overall, our analysis indicates a high positive detection rate of SARS-CoV-2 RNA in plasma (44%), mostly in the early stage of infection before seroconversion. Notably, viral clearance was significantly associated with the detection of SARS-CoV-2 antibodies. Based on the present data, our hypothesis is that the early stage of SARS-CoV-2 infection is characterized by a primary RNAemia, as a potential manifestation of a systemic infection. Additionally, the viral load in plasma seems to be associated with a worse disease outcome.

## Figures and Tables

**Table 1:** Patient characteristics according to disease severity of coronavirus disease 2019 (COVID-19) and laboratory parameters at time of first positive respiratory RT-PCR after hospital admission.

	Mild or Moderate Cases, *n* = 6 (19%)	Severe Cases, *n* = 14 (44%)	Critical Cases, *n* = 12 (37%)	*p* Value	Adjusted *p* Value
Age in years, median (IQR)	60 (55–65)	55 (50–66)	67 (57–76)	0.273	
Female, *n* (%)	5 (83)	6 (43)	3 (25)	0.063	
Detection of viremia, *n* (%)	2 (33)	5 (36)	7 (58)	0.434	
Fatal outcome, *n* (%)	1 (17)	0 (0)	5 (42)	0.025 *	
Leucocytes, median (IQR)	5.06 (2.79–7.02)	5.72 (3.82–7.43)	7.49 (6.71–10.06)	0.170	
Lymphocytes, median (IQR)	0.9 (0.62–1.44)	0.89 (0.80–1.37)	0.78 (0.54–0.89)	0.461	
CRP, median (IQR)	15 (9–80)	102 (60–193)	215 (101–263)	0.009 *	m/s: 0.076 m/c: 0.010 s/c: 0.076
IL-6, median (IQR)	26 (10–30)	52 (30–90)	156 (48–319)	0.005 *	m/s: 0.035 m/c: 0.015 s/c: 0.035

CRP, C-reactive protein; IL-6, interleukin 6; RT-PCR, real-time PCR; IQR, Interquartile range; * *p* < 0.05.

**Table 2:** Random intercept models with individual patients as random effect to assess the association of fixed effects on of severe acute respiratory syndrome coronavirus 2 (SARS-CoV-2) Ct (cycle threshold) values in respiratory and plasma samples. CI, confidence interval; Ct, cycle threshold; * *p* < 0.005.

		SARS-CoV-2 Ct in Respiratory Samples			SARS-CoV-2 Ct in Plasma Samples		
Random Effect	Patient	Estimate	95% CI	*p* Value	Estimate	95% CI	*p* Value
Fixed Effects	IgG titer	0.50	0.21; 0.81	0.002 *	0.38	0.22; 0.55	<0.001 *
	Male Female	1 0.08	−3.59; 3.73	0.968	1 −0.76	−2.64; 1.09	0.457
	Age in years	−0.10	−0.22; 0.02	0.128	−0.04	−0.10; 0.03	0.307
	Days after onset of symptoms or first positive PCR	0.26	0.16; 0.34	<0.001 *	0.06	−0.01; 0.11	0.017 *
	Severity: mild/moderate Severe Critical	1 1.54 −3.55	−3.40; 6.42 −8.95; 1.80	0.565 0.232	1 −3.26 −4.39	−5.83; −0.75 −7.19; −1.61	0.024 * 0.007 *

## Supplementary Materials

The following are available online at https://www.mdpi.com/1999-4915/12/9/1045/s1, Table S1: Patient characteristics of 32 hospitalized COVID-19 patients and laboratory results on the day of first positive respiratory PCR result after admission, Figure S1: Individual longitudinal development of virological results of 32 patients with COVID-19 infection during hospital admission, Figure S2: Grouped longitudinal virological parameters of 32 hospitalized COVID-19 patients with and without viremia.
