# Supplementary material for: RNAemia Corresponds to Disease Severity and Antibody Response in Hospitalized COVID-19 Patients
Source: Viruses. 2020 Sep 18;12(9):1045. doi: 10.3390/v12091045 (PMC7551174; doi:10.3390/v12091045)
Supplement: Supplementary file 1 [file viruses-12-01045-s001.pdf]

viruses

MDPI

Supplementary

RNAemia Corresponds to Disease Severity and Antibody Response in Hospitalized COVID-19 Patients

Kirsten Alexandra Eberhardt [1,†], Charlotte Meyer-Schwickerath [2,†], Eva Heger [3], Elena Knops [3], Clara Lehmann [2,4,5], Jan Rybniker [2,4,5], Philipp Schommers [2,4], Dennis A. Eichenauer [2,6,7], Florian Kurth [1], Michael Ramharter [1], Rolf Kaiser [3], Udo Holtick [2,6], Florian Klein [3,4,5], Norma Jung [2] and Veronica Di Cristanziano [3,*]

1 Department of Tropical Medicine, Bernhard Nocht Institute for Tropical Medicine and I. Department of Medicine, University Medical Center Hamburg-Eppendorf, 20251 Hamburg, Germany; k.eberhardt@bnitm.de (K.A.E); florian.kurth@bnitm.de (F.K.); ramharter@bnitm.de (M.R.)
2 Department I of Internal Medicine, Medical Faculty and University Hospital Cologne, University of Cologne, 50937 Cologne, Germany; charlotte.meyer-schwickerath@uk-koeln.de (C.M.-S); clara.lehmann@uk-koeln.de (C.L.); jan.rybniker@uk-koeln.de (J.R.); philipp.schommers@uk-koeln.de (P.S.); dennis.eichenauer@uk-koeln.de (D.A.E.); udo.holtick@uk-koeln.de (U.H.); norma.jung@uk-koeln.de (N.J.)
3 Institute of Virology, Faculty of Medicine and University Hospital of Cologne, University of Cologne, 50935 Cologne, Germany; eva.heger@uk-koeln.de (E.H.); elena.knops@uk-koeln.de (E.K.); rolf.kaiser@uk-koeln.de (R.K.); florian.klein@uk-koeln.de (F.K.)
4 German Center for Infection Research (DZIF), Partner Site Bonn-Cologne, 50937 Cologne, Germany
5 Center for Molecular Medicine Cologne, University of Cologne, 50937 Cologne, Germany
6 Center of Integrated Oncology ABCD, University Hospital of Cologne, 50937 Cologne, Germany
7 Intensive Care Program, Department I of Internal Medicine, University Hospital of Cologne, 50937 Cologne, Germany
* Correspondence: veronica.di-cristanziano@uk-koeln.de; Tel.: +49-221-478-85828
† These authors contributed equally to this work.

Abstract: Severe acute respiratory syndrome coronavirus 2 (SARS-CoV-2) represents a global health emergency. To improve the understanding of the systemic component of SARS-CoV-2, we investigated if viral load dynamics in plasma and respiratory samples are associated with antibody response and severity of coronavirus disease 2019 (COVID-19). SARS-CoV-2 RNA was found in plasma samples from 14 (44%) out of 32 patients. RNAemia was detected in 5 out of 6 fatal cases. Peak IgG values were significantly lower in mild/moderate than in severe (0.6 (interquartile range, IQR, 0.4–3.2) vs. 11.8 (IQR, 9.9–13.0), adjusted $p = 0.003$) or critical cases (11.29 (IQR, 8.3–12.0), adjusted $p = 0.042$). IgG titers were significantly associated with virus Ct (Cycle threshold) value in plasma and respiratory specimens ((ß = 0.4, 95% CI (confidence interval, 0.2; 0.5), $p < 0.001$ and ß = 0.5, 95% CI (0.2; 0.6), $p = 0.002$). A classification as severe or a critical case was additionally inversely associated with Ct values in plasma in comparison to mild/moderate cases (ß = −3.3, 95% CI (−5.8; 0.8), $p = 0.024$ and ß = −4.4, 95% CI (−7.2; 1.6), $p = 0.007$, respectively). Based on the present data, our hypothesis is that the early stage of SARS-CoV-2 infection is characterized by a primary RNAemia, as a potential manifestation of a systemic infection. Additionally, the viral load in plasma seems to be associated with a worse disease outcome.

Keywords: SARS-CoV-2; humoral response; seroconversion; dynamics; viral load; blood; viremia; antibodies

Table S1. Patient characteristics of 32 hospitalized COVID-19 patients and laboratory results on the day of first positive respiratory PCR result after admission.

Patient ID	Age	Sex	Underlying pathologies	Severity of CoVID-19	CRP mg/L (<5)	Leucocytes abs. x10⁹/L (4.4–11.3)	Lymphocytes abs. x10⁹/L (1.26–3.35)	IL-6 ng/L (<8)	D-Dimer mg/L <0.5
1	66	F	Auto-hematopoietic stem cell transplantation (HSCT) due to multiple myelom	mild	112.8	0.89	0.05	31	1.05
2#	51	F	Acute myeloid leukemia after allo-HSCT	severe	218.9	0.84	0.31	276	12.62
3#	52	M	Rheumatoid arthritis, chronic kidney failure, adrenal tumor, recurrent thrombosis	severe	93.3	6.28	0.89	49	1.82
4#*	77	F	Secondary acute myeloid leukemia from myelodysplasia	critical ill	119.6	0.23	NM	36	ND
5#*	88	F	Brain tumor	mild	0.6	13.5	0.86	2	ND
6	52	M	Chronic lymphocytic leukemia with hypogammaglobulinemia	critical ill	27.2	28.57	ND	ND	ND
7	31	F	Ulcerative colitis	severe	228.8	1.76	0.29	76	2.54
8#*	65	M	Glioblastom, type 2 diabetes mellitus	critical ill	44.5	7.34	ND	24	ND
9	53	F	Liver transplantation, chronic renal failure, polyneuropathy	mild	12.7	2.16	0.55	28	ND
10	50	F	HIV infection under antiretroviral therapy	severe	110.6	7.74	0.9	27	1.06
11	30	F	Ciliary dyskinesia	moderate	18.1	5.45	1.87	5	0.64
12#	53	M	Arterial hypertension, type C Gastritis, spina bifida occulta	critical ill	345.2	16.58	1.4	389	ND
13	76	F	Arterial hypertension, atrial fibrillation, cholecystolithiasis	severe	17	3.77	1.91	30	0.47
14	80	F	Pulmonary artery embolism, arterial hypertension, Basedow´s disease	critical ill	231.6	7.78	0.57	152	ND
15#*	62	F	Chronic obstructive pulmonary disease, bronchial asthma, arterial hypertension, adipositas, sleep apnea	critical ill	276.1	6.67	0.79	249	ND
16*	70	M	Intervertebral disc prolapse	critical ill	258	9.22	0.78	3100	ND
17	76	M	Prostata und kindey cancer, adrenal adenoma, sigma elongatum, colon polyps, arterial hypertension	critical ill	223	12.56	0.96	59	ND
18#	76	M	Atrial fibrillation	severe	30	5.62	0.85	59	0.99
19#	68	M	Chronic venous insufficiency	severe	6.6	3.98	1.41	19	ND
20	56	M	Sinubronchial syndrome	severe	68.7	6.51	1.37	6	0.48
21#	76	M	Internal carotid artery stenosis, arterial hypertension, chronic kidney failure, psychosis	critical ill	25.2	3.86	0.19	33	0.89
22	59	F	Bronchial asthma, depression	severe	195.7	8.4	1.11	55	0.99

23#	85	F	Heart failure, hemodialysis, type 2 diabetes mellitus, polyneuropathy	severe	58.8	3.1	ND	112	2.55
24	38	F	None	severe	65.2	4.9	0.86	33	0.41
25#	62	M	Sleep apnea	moderate	8.3	4.67	0.93	25	0.37
26#*	52	M	Chronic hepatitis B virus infection	critical ill	203.9	7.64	0.87	156	ND
27	54	M	None	severe	183	9.06	0.8	94	0.95
28	64	M	None	critical ill	206.8	7.33	0.43	167	4.18
29	58	M	None	severe	229.4	5.82	0.7	31	6.87
30	50	M	Arterial hypertension	severe	124.6	19.13	11.59	94	0.46
31	57	F	None	moderate	100.4	7.54	1.62	48	0.59
32#	58	M	None	critical ill	462.7	6.72	ND	548	4.32

patient with RNAemia; * exitus letalis; ND= not done; NM= not measurable.

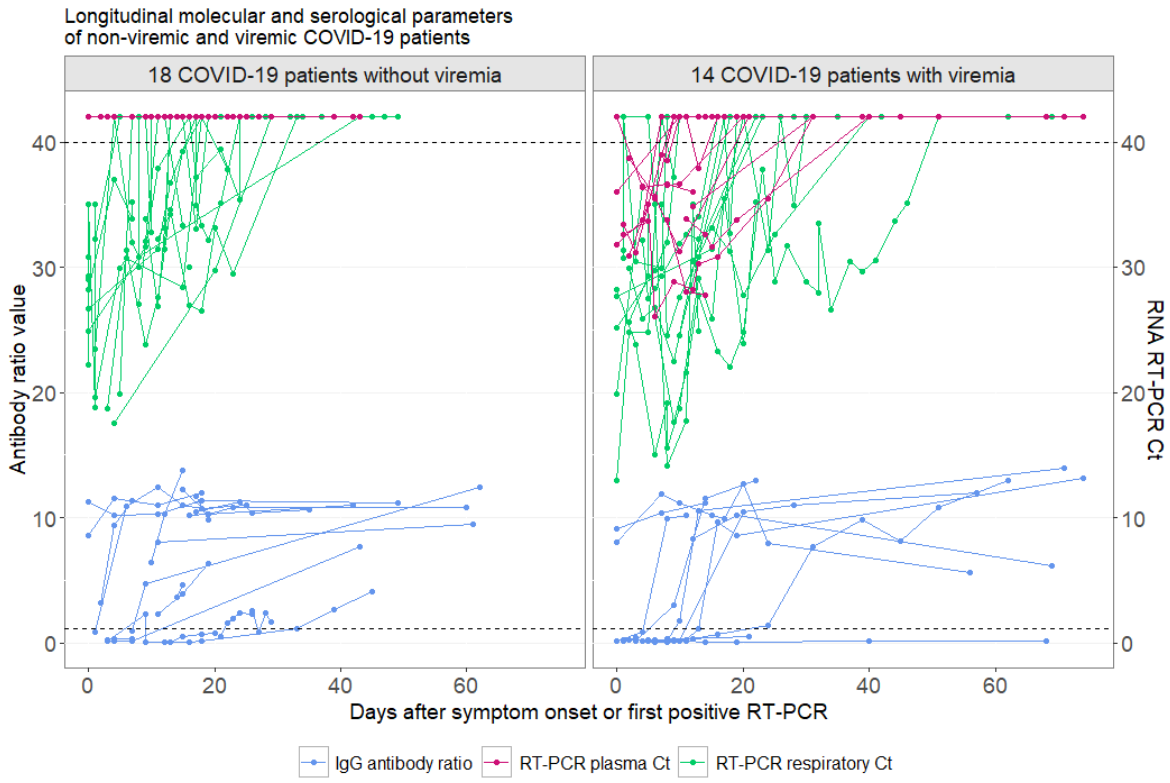

Figure S1. Grouped longitudinal virological parameters of 32 hospitalized COVID-19 patients with and without viremia. Longitudinal dynamics of SARS-CoV-2 RNA Ct values in respiratory and plasma samples and IgG. Negative PCR results are displayed above the detection Ct (Cycle threshold) of 40. Dashed horizontal lines display the detection threshold of SARS-CoV-2 RNA Ct values and the antibody cut-off of seroconversion for the assay used. RT-PCR, real-time PCR.

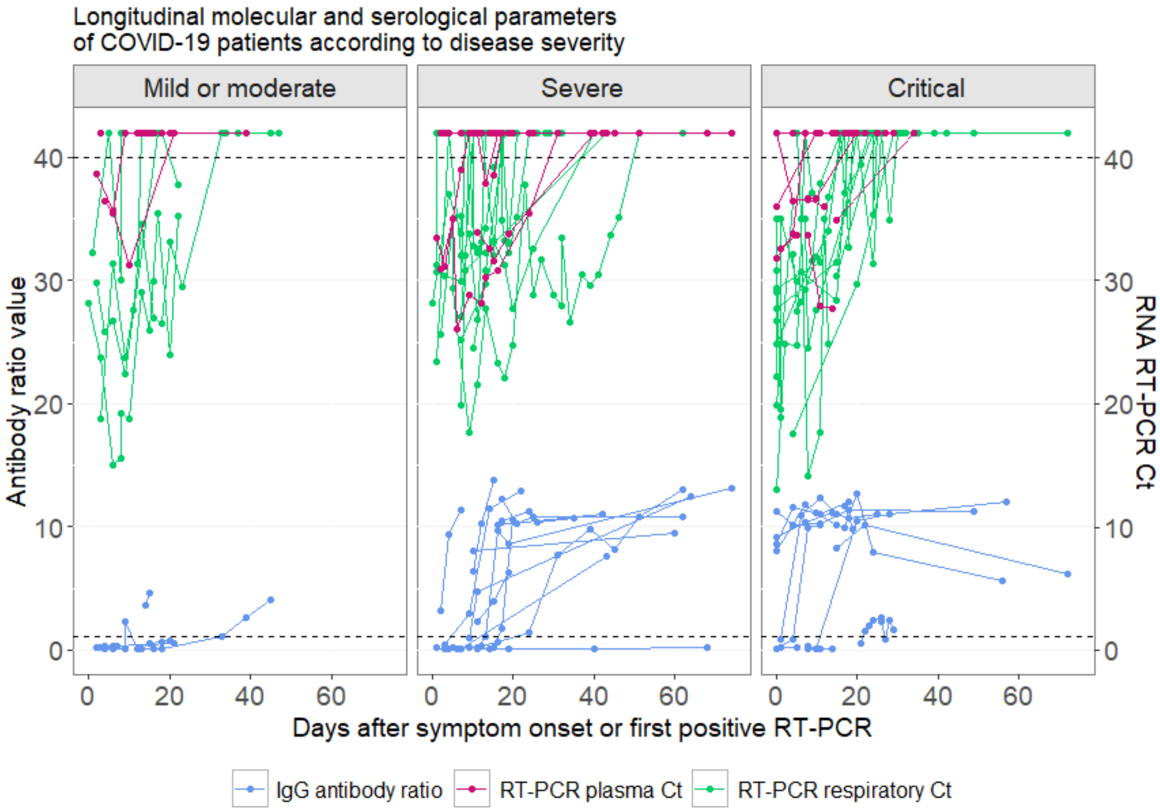

Figure S2. Grouped longitudinal virological parameters of 32 hospitalized COVID-19 patients according to disease severity. Longitudinal dynamics of SARS-CoV-2 RNA Ct values in respiratory and plasma samples and IgG. Negative PCR results are displayed above the detection Ct (Cycle threshold) of 40. Dashed horizontal lines display the detection threshold of SARS-CoV-2 RNA Ct values and the antibody cut-off of seroconversion for the assay used. RT-PCR, real-time PCR.
